# Complete coding region sequence analyses and antigenic characterization of emerging lineage G-IX of foot- and-mouth disease virus serotype Asia1

**DOI:** 10.1080/01652176.2024.2367215

**Published:** 2024-06-21

**Authors:** Manoranjan Rout, Shyam Singh Dahiya, Saravanan Subramaniam, Ramakant Acharya, Reshama Samanta, Jitendra Kumar Biswal, Jajati Keshari Mohapatra, Rabindra Prasad Singh

**Affiliations:** International Centre for Foot and Mouth Disease, ICAR-National Institute on Foot and Mouth Disease, Bhubaneswar, India

**Keywords:** Foot and mouth disease virus, serotype Asia 1, group-IX, India, vaccine strain, Emergence, Lineage, ORF

## Abstract

Foot-and-mouth disease Virus (FMDV) serotype Asia1 is prevalent in the Indian subcontinent, with only G-III and G-VIII reported in India until 2020. However, in 2019, a novel genetic group within serotype Asia1, designated as G-IX, emerged in Bangladesh, followed by its detection in India in 2020. This report presents analyses of the complete coding region sequences of the G-IX lineage isolates. The length of the open reading frame (ORF) of the two G-IX isolates was 6990 nucleotides without any deletion or insertion. The G-IX isolates showed the highest sequence similarity with an isolate of G-III at the ORF, L, P2, and P3 regions, and with an isolate of G-VIII at the P1 region. Phylogenetic analysis based on the capsid region (P1) supports the hypothesis that G-VIII and G-IX originated from a common ancestor, as speculated earlier. Further, VP1 region-based phylogenetic analyses revealed the re-emergence of G-VIII after a gap of 3 years. One isolate of G-VIII collected during 2023 revealed a codon insertion in the G-H loop of VP1. The vaccine matching studies support the suitability of the currently used Indian vaccine strain IND63/1972 to contain outbreaks due to viruses belonging to G-IX.

## 1. Introduction

The extensive genetic diversity and ongoing evolution of the foot-and-mouth disease virus (FMDV) remain significant challenges for the livestock economies of numerous countries. FMDV, belonging to the *Aphthovirus* genus within the *Picornaviridae* family, consists of seven distinct serotypes (O, A, C, Asia 1, SAT 1-3), each immunologically distinct and lacking cross-protection. These serotypes are further classified into various topotypes, lineages, sub-lineages, and strains, reflecting the complex genetic landscape of the virus. Among these, Serotype Asia1 is genetically and antigenically the least divergent, with a single topotype primarily restricted to Asia (Brito et al. 2018). The RNA virus’s fast mutation rate and quasi-species nature have led to the continuous evolution of genetically and antigenically diverse strains within each serotype (Domingo et al. 2003). The FMDV genome organization consists of a single open reading frame (ORF) that encodes a polyprotein, flanked by a long 5′-untranslated region (UTR) and a short 3’UTR with a poly(A) tail. FMD is endemic in India, with prevalent serotypes O, A, and Asia1. Several factors, including high densities of susceptible ruminants and swine populations, diverse agroclimatic regions, and various animal husbandry techniques in mixed livestock farming scenarios, significantly contribute to the maintenance and emergence of novel FMDV variants in India.

FMDV serotype Asia 1 was initially reported in India during 1951–52 (Dhanda et al. 1957). Historically, the primary endemic region for FMDV serotype Asia 1, before 2003, was mainly southern Asia, where outbreaks were regularly documented. However, post-2003, serotype Asia 1 viruses expanded northward, leading to widespread outbreaks of FMD across Asia (Valarcher et al. 2009). Serotype Asia1 isolates from various Asian countries were categorized into nine distinct genetic groups designated as G-I to G-IX (Valarcher et al. 2009; Jamal et al. 2011; Subramaniam et al. 2013; Ali et al. 2019). These classifications were based on analyses of the complete VP1 region. In India, three groups, namely G-III, G-VIII, and G-IX, have been identified. Group III was in circulation for a shorter period during 2003–2004. Since 2005, FMD outbreaks in Asia1 serotype have been attributed to G-VIII, which remained the sole genetic group circulating in India between 2005 and 2019 (Subramaniam et al. 2013). In January 2020, the new genetic group, G-IX, was documented in an organized farm in southern India, having been first identified in Bangladesh in 2019 (Ali et al. 2019; Subramaniam et al. 2020).

The high contagiousness and the presence of multiple serotypes and variants make FMD challenging to control. Continuous epidemiological investigation is crucial for implementing effective vaccination programs and control measures. Globally, phylogenetic analysis based on nucleotide sequence data, particularly of the VP1 region, has been widely adopted for molecular epidemiological surveillance. However, complete genome sequence analysis is essential for exploring changes in the genetic features of novel lineages. Additionally, *in vitro* 2-dimensional virus neutralization test (2d-VNT) has been utilized to evaluate the antigenic relationships of circulating and emergent field strains with the in-use vaccine strain. This method aids in determining the suitability of vaccine strains and enables the selection of alternative strains if necessary. The antigenic relationship of the newly emerging G-IX strains with the Indian vaccine strain IND63/1972 has not yet been reported. In light of this, the present study was designed to identify the prevalent serotype Asia1 genetic groups in India since 2020 and to assess their antigenic relatedness with the Indian vaccine strain. Additionally, the complete coding region sequence of the emerging G-IX lineage is characterized and reported here for the first time.

## 2. Materials and methods

In the current study, seven isolates of FMDV serotype Asia1, which were collected between 2020 and 2023 were characterized (Table 1). The VP1 region was sequenced for five isolates, and molecular epidemiological analyses were performed on them. For two of these five isolates that belonged to G-IX genetic group, the complete coding region sequence has been determined. For the remaining two isolates, only vaccine matching studies were done, and unfortunately, sequencing was not possible.

**Table 1.** History of field isolates of FMDV serotype Asia1 characterized in this study. These isolates were collected from three different states of India during 2020–23.

S No	Isolate ID	Species	Date of isolation	Place of collection	Analyses
1	As/ICFMD1/2020	Cattle	January 2020	Kattupakkam, Tamil Nadu	Vaccine matching analyses
2	As/ICFMD4/2020	Cattle	January 2020	Kattupakkam, Tamil Nadu	Complete coding region, VP1 region & Vaccine matching analyses
3	As/ICFMD8/2020	Cattle	January 2020	Kattupakkam, Tamil Nadu	Vaccine matching analyses
4	As/ICFMD120/2021	Cattle	2021	Khansahib, Jammu & Kashmir	Complete coding region, VP1 region & Vaccine matching analyses
5	As/ICFMD274/2022	Cattle	27/07/2022	Sangam, Jammu & Kashmir	VP1 region analyses
6	As/ICFMD277/2022	Cattle	27/07/2022	Sangam, Jammu & Kashmir	VP1 region analyses
7	As/ICFMD 45/2023	Cattle	24/01/2023	Dharampura, Gujarat	VP1 region & Vaccine matching analyses

For sequencing of VP1 region, total RNA was extracted from serotype confirmed five infected BHK-21 cell culture supernatants of early passage level (P3-P5) using RNeasy Mini Kit (Qiagen, Germany) following the manufacturer’s instructions. Reverse transcription was carried out using MMLV reverse transcriptase (Promega, USA) and NK61 (5′-GACATGTCCTCCTGCATCTG, negative-sense, location 2B, 58-77) primer (Knowles and Samuel 1994). The PCR amplification of VP1/1D gene was carried out with the primer combination of 1C505 (5′-TACACTGCTTCTGACGTGGC, Asia 1 serotype specific, positive-sense, location VP3, 505-524) (Knowles and Samuel 1994) and NK61 using *pfu* DNA polymerase (Fermentas, USA). The PCR conditions used were essentially as described earlier (Subramaniam et al. 2020). The PCR products were purified using QIA quick Gel Extraction Kit (Qiagen, Germany) and then the amplicons were sequenced using an ABI 3130 Genetic Analyzer (Applied Biosystems, USA).

The complete coding region sequences were determined for two isolates (ICFMD4/2020 and ICFMD120/2021) of G-IX that were collected from the field outbreaks during the consecutive years 2020 and 2021. The RNA obtained from infected BKH-21 cells at passage 4 were sequenced using an Illumina Novaseq6000 platform (Illumina, USA) through outsourcing (Eurofins Genomics Pvt Ltd, India, Bengaluru). Library preparation was performed following the IlluminaTruSeq RNA library protocol outlined in the ‘TruSeq RNA Sample Preparation Guide’ (Illumina). The prepared library was quantified using Qubit and validated for quality by running an aliquot on High Sensitivity Bioanalyzer Chip (Agilent). The Illumina paired end (2 × 150 bp) raw reads were quality checked using FastQC (Andrews, 2010). The high-quality reads of both samples were aligned to the reference sequence using BWA MEM (version 0.7.17) and consensus sequence(s) was extracted using Samtools-mpileup.

For phylogenetic analyses, in addition to the VP1 sequences of 5 strains generated in this study, additional VP1 sequences were retrieved from the GenBank and the Institute Genetic Database. The sequences were aligned using the MUSCLE tool (Edgar, 2004). The mean and pairwise divergence were then computed. To assess the evolutionary relationships among FMDV isolates, phylogenetic trees were inferred by the Maximum Likelihood (ML) method based on the nucleotide alignment of the VP1, ORF, L, P1, P2 and P3 sequences using the MEGA software v. 11 (Tamura et al. 2021). The ML phylogeny was produced under the nucleotide substitution model with rate variation following a gamma distribution as determined by the model finder, and the robustness of the tree topology was assessed by bootstrap analysis with 1000 iterations. Using time-stamped sequence data with a relaxed and an uncorrelated lognormal clock under the Bayesian Markov chain Monte Carlo (MCMC) method, BEAST version 1.10.4 (Suchard et al. 2018) was used to estimate the rates of evolutionary change (nucleotide substitutions per site per year), and mutation at different codon positions.

To ascertain the positive selection pressure at specific codon locations, two likelihood approaches: the fixed effects likelihood (FEL) method and the single likelihood ancestor counting (SLAC) method (Kosakovsky Pond and Frost 2005), and a Bayesian strategy known as FUBAR (Murrell et al. 2013) were utilised. The level of selection pressure was calculated as the ratio of non-synonymous (dN) to synonymous (dS) substitutions per site (ratio: dN/dS). Positive selection is strongly implied when the posterior probability is more than 0.9 for FUBAR and less than 0.1 for SLAC. The codon locations that were the target of episodic diversifying selection were determined using the Mixed Effects Model of Evolution (MEME) (Murrell et al. 2012). Significant evidence of selection was acknowledged at p-values less than 0.05. The Datamonkey webserver (Weaver et al. 2018) was used for all of the analyses. The complete coding region sequence alignment was examined for any signs of recombination using the RDP5 software v. 5.30 (Martin et al. 2020). The analysis was carried out using various methods available and their default parameters. Recombination events were only deemed proven if they were identified by all the seven available algorithms (RDP, Geneconv, BootScan, MaxChi, Chimaera, SiScan, and 3Seq) with default values. RDP, MaxChi, and Chimaera use a sliding window to screen for recombination in polymorphic sites for every potential triplet of a sequence. GENECONV looks for commonalities between aligned pairwise locations to identify potential gene conversion events. 3SEQ is a non-parametric approach that finds important breakpoint regions using a ranked clustering statistic. Bootscan projects conflicting phylogenetic signals in different regions of an alignment, while SiSscan analyses changes in phylogenetic signals in gene sequences that arise from recombination.

Two-dimensional virus-neutralization test (2D-VNT) was performed as described earlier (Rweyemamu et al. 1978) using bovine vaccinate serum (BVS) against the currently used vaccine strain (IND63/72) for five isolates. BHK 21 cells were used as indicator system in neutralization test. The BVS was obtained from the Serum Repository of ICAR-NIFMD, Bhubaneswar, India. Before testing, the sera were inactivated at 56 °C for 30 min in a water bath followed by use in 2D-VNT. The antibody titre was determined as the reciprocal of the last dilution of serum that neutralised 100TCID_50_ in 50% of the wells. The relationship value (r1-value) was calculated as a ratio of antibody titres against heterologous field isolates to those against the homologous vaccine strain, averaged from the two separate runs. An r1-value of >0.3 indicates that there is sufficient antigenic similarity between the vaccine strain and a field isolate. Conversely, an antigenic divergence is indicated by the r1-value of <0.3 (Rweyemamu, 1984).

## 3. Results

### 3.1. Phylogenetic clustering

**Figure 1.**
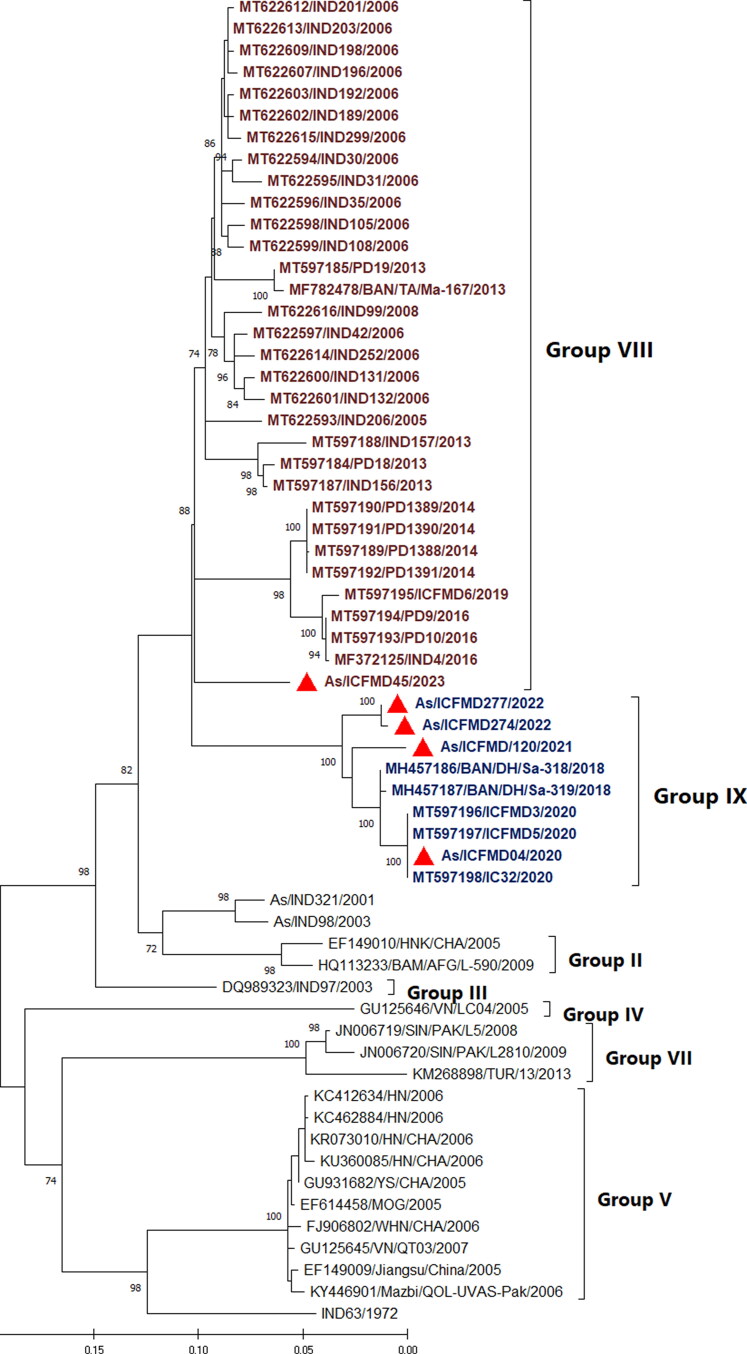
Maximum Likelihood phylogenetic tree reconstructed based on the VP1 coding region, depicting different genetic groups of serotype Asia1. The isolates sequenced in this study are marked by filled red triangles. The figure indicates the percentage of bootstraps (out of 1000) supporting the corresponding clade. Branch lengths are measured in substitutions per site. Four out of five isolates sequence determined in this study clustered within G-IX.

Phylogenetic analyses conducted in this study revealed that four out of the five Asia1 isolates sequenced belonged to the newly identified genetic group G-IX (Figure 1). Among these four isolates, three were collected from Jammu and Kashmir, the northernmost region of the country, in 2021 and 2022, while one isolate was from the state of Tamil Nadu, the southernmost region, in 2020. Additionally, one isolate collected from Gujarat in 2023 clustered separately with G-VIII.

### 3.2. Detection of indel variant

**Figure 2.**
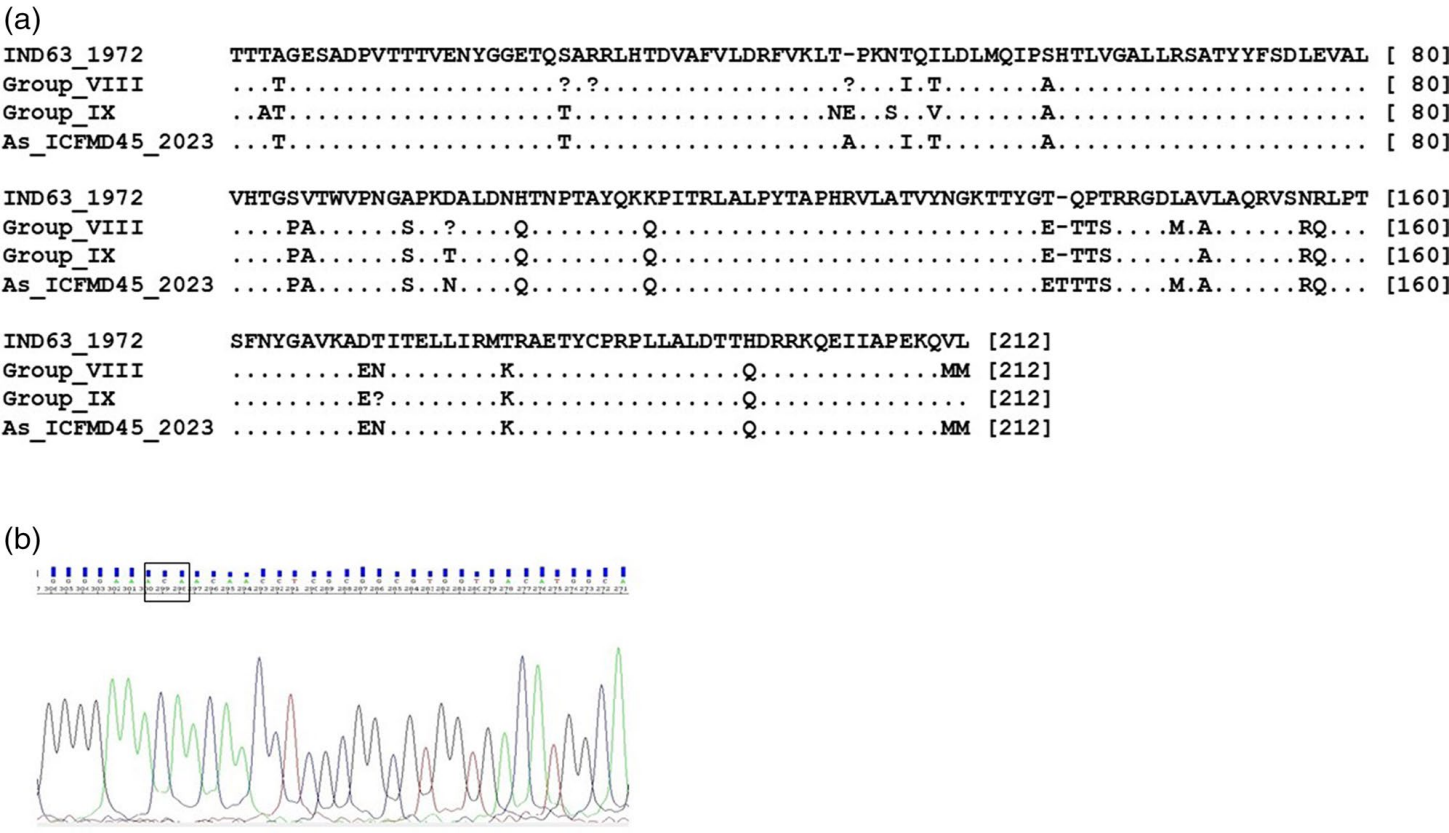
2a. Alignment of deduced VP1 amino acid sequences of serotype Asia strains belonging to G-VIII, G-IX, and an isolate with an insertion, with that of the vaccine strain IND63/1972. A dot indicates similarity with the vaccine strain, while variations are shown as single-letter amino acid codes. 2b. Chromatogram displaying the ‘ACA’ codon insertion in the isolate As/ICFMD45/2023, resulting in the insertion of the amino acid ‘Threonine’ at the 139th position of VP1.

The consensus length of the VP1 region of serotype Asia1 is 633 nucleotides (nts), encoding a 211 amino acid (aa) long protein (Figure 2a). In the current investigation, an Asia1 field isolate (ICFMD45/2023) from Gujarat state, collected in 2023, displayed an amino acid insertion in the βG-βH loop of VP1 (Figure 2b).

### 3.3. Comparisons of complete coding region

The two complete genome sequences of FMDV serotype Asia1 generated in this study for isolates ICFMD4/2020 and ICFMD120/2021 have been deposited in GenBank under accession numbers OR916275 and OR916276, respectively. The complete open reading frame (ORF) sequences of these two serotype Asia 1/G-IX isolates were aligned and compared with 23 sequences available in GenBank (Table 2). The ORF of the two G-IX isolates was 6990 nts in length, encoding 2329 aa long polyprotein. It includes the L protein (201 aa), four structural proteins [VP4 (85 aa), VP2 (219 aa), VP3 (218 aa), and VP1 (211 aa)], and eight non-structural proteins [2A (16 aa), 2B (154 aa), 2 C (318 aa), 3 A (153 aa), 3B (71 aa), 3 C (213 aa), and 3D (470 aa)]. No indels were observed in the genome of G-IX viruses. Unfortunately, the untranslated region (UTR) region sequence flanking the ORF could not be generated in this study.

**Table 2.** Details of the FMDV serotypes Asia 1 isolates used for comparison of complete ORF. The sequences were obtained from NCBI database.

Sl No	Sample ID	Place of collection	Year of collection
1	MF782478	Bangladesh	2013
2	MF372125	India	2016
3	DQ989323	India	2002
4	MN366244	Bangladesh	2018
5	HQ113233	Afghanistan	2009
6	EF149010	China	2005
7	KY825721	China	2005
8	JN006720	Pakistan	2009
9	MN853346	Iran	2017
10	KM268898	Turkey	2013
11	JN006719	Pakistan	2008
12	GU125646	Viet Nam	2005
13	GU125645	Viet Nam	2007
14	FJ906802	China	2006
15	HQ631363	China	2006
16	KY446901	Pakistan	2006
17	EF149009	China	2005
18	EF614458	Mongolia	2005
19	GU931682	China	2005
20	KU360085	China	2015
21	KR073010	China	2006
22	KC462884	China	2006
23	KC412634	China	2006

The G-IX isolates exhibited nt differences ranging from 7.8% to 8.4% with G-II, 7.8% to 8.3% with G-III, 10.3% to 10.6% with G-V, 9.4% to 10.3% with G-VII, and 9.0% to 10.3% with G-VIII, at the ORF level. The mean distance at the ORF level varied from 7.9% to 10.6% between different genetic groups. Overall, the mean nt distance among the groups compared in this study was 8%. Two group-specific signatures were observed in G-IX isolates at positions 43 (T→N) of VP1 and 38 (V→M) of VP2. When the ORF was divided into the L, P1, P2, and P3 regions, the calculated mean nt distances were 10%, 11%, 6%, and 6%, respectively, among the Asia1 isolates. The P2 and P3 regions, which code for non-structural proteins including polymerase and protease, were more conserved. Comparing the nt identities of different regions of the G-IX genome with other genetic groups of the Asia1 serotype, the G-IX isolate showed the highest sequence homology of 92.3%, 93.9%, 92.8%, and 93.9% at the ORF, L, P2, and P3 regions, respectively, with an isolate of G-III. It also exhibited 90.5% sequence homology with an isolate of G-VIII at the P1 region. In contrast, the G-IX isolate had a minimum nt sequence homology of 89.5%, 86.2%, and 91.1% at the ORF, L, and P3 regions, respectively, with an isolate of G-IV. Additionally, it showed 91.4% sequence homology with an isolate of G-II at the P2 region and 84.4% with an isolate of G-V at the P1 region.

The mean rate of evolutionary change in FMDV Asia1 was estimated to be 3.872 × 10^−3^ substitutions per site per year for the ORF (Table 3). When comparing the substitution rates of the L, P1, P2, and P3 regions, the estimates decreased in the following order: P1, L, P2, and P3. The faster evolutionary rate in the L region is mainly attributed to the high variability in the region between two start codons. Regarding the relative nt substitution rates at all three codon positions in the L, P1, P2, and P3 regions, mutations were more frequent at the third codon position compared to the first and second positions. Interestingly, a region-wise difference was observed, with the L region followed by P1 having a higher mutation rate in codon positions 1 and 2, while the P2 region followed by P3 had a higher mutation rate in codon position 3.

**Table 3.** The evolutionary rates, and mutation rates at three codon sites of FMDV serotype Asia1 estimated using Bayesian Markov chain Monte Carlo analysis.

Region	Coefficient of variation	Evolutionary rate (95% HPD)	CP1	CP2	CP3
ORF	0.545	3.872 × 10^-3^ (2.064 × 10^-3^ –5.777 × 10^-3^)	–	–	–
L	0.998	6.793 × 10^-3^ (3.356 × 10^-3^ – 1.1 × 10^-2^)	0.439	0.198	2.364
P1	0.680	7.316 × 10^-3^ (4.434 × 10^-3^ –1.105 × 10^-2^)	0.331	0.177	2.492
P2	0.704	2.312 × 10^-3^ (5.742 × 10^-4^ –4.264 × 10^-3^)	0.247	0.133	2.620
P3	0.545	2.596 × 10^-3^ (9.058 × 10^-4^ –4.295 × 10^-3^)	0.295	0.137	2.568

The complete ORF, along with four segments of the ORF, underwent Bayesian analysis. The P1 region, which codes for structural proteins, exhibited the highest evolutionary rate.

### 3.4. Antigenic site comparison

For analysing the variation in the antigenic sites, the capsid region sequences of G-IX isolates were compared with that of currently used Indian vaccine strain IND63/1972. In G-IX isolates, antigenic site I showed consensus changes at positions VP1-140 (P→T), and in two isolates, T→S changes at position VP1-141, similar to those observed in G-VIII (Subramaniam et al. 2015). Similarly, at site II, a consensus variation at position VP2-74 (S→A) was found in G-IX and G-VIII. Furthermore, antigenic site IV was found to be perfectly conserved, and site V showed replacement at position VP3-218 (R→Q) in relation to the vaccine strain in one isolate.

The RGD motif and two L residues located at positions +1 and +4 downstream of the RGD, which have an essential role in cell recognition are fully conserved (Mateu et al. 1996). Group-specific changes were reported in this motif by Jamal et al. (2011). For instance, RGDLAAI, RGDMAAL, and RGDLAAI/V were found in G-I, G-VI, and G-VII isolates, respectively. Even a variety of different motifs were reported in the Group-II viruses. In the three genetic groups reported in India, two different motifs were found: RGDMAAL in G-III and G-VIII and RGDLAAL in G-IX isolates, thereby maintaining at least one of the two L residues downstream of the RGD motif. Three of the four G-IX isolates and one G-VIII isolate analyzed in the present study had RGDLAAL and RGDMAAL motifs, respectively.

### 3.5. Recombination analyses

Recombination analyses of the 25 ORF revealed a mosaic pattern in serotype Asia1 isolates. Recombination events were considered only when detected by all seven methods available in RDP. Although four events of recombination were detected (data not shown), three events could not be confirmed as the parent and recombinant sequences did not overlap in time and space. In the one confirmed recombinant event, the recombinant isolate JN006720/SIN/PAK/L2810/2009 belongs to G-VII, with major and minor parents HQ113233 (G-II) and JN006719 (G-VII), respectively. Earlier, this isolate was identified as an inter-serotypic recombinant of serotype Asia1 and A (Jamal et al. 2011). None of the isolates of G-IX analyzed in the current study showed any evidence of recombination.

### 3.6. Selection pressure analyses

Genome-wide selection pressure analyses of 25 isolates revealed a dN/dS ratio of 0.0679, indicating that evolution is strongly influenced by negative selection pressure. Site-specific selection analyses identified two codon positions (72 and 291) in 3D as being under pervasive positive selection pressure, as determined by SLAC (*p* < 0.1) and FUBAR (posterior probability > 0.9) with statistical significance. FUBAR, with a posterior threshold of 0.9, achieves very low false-positive rates on data simulated under neutrality (Murrell et al. 2013). Additionally, one site (172) in VP2 was identified by FUBAR alone as being under pervasive positive selection pressure. The MEME likelihood approach was employed to identify sites under episodic positive selection, revealing twenty-eight codon positions (L-112, VP4-70, VP2-2, 63, 95, 100, 172, 173, VP3-173, VP1-5, 6, 44, 47, 144, 146, 154, 2 C-66, 67, 71, 76, 78, 84, 92, 3 A-167, and 3D-72, 221, 242, 291) that were under episodic positive selection pressure (*p* < 0.05).

### 3.7. Vaccine matching analyses

To determine the antigenic relationship of As/G-IX isolates, recently isolated serotype Asia 1 field viruses underwent vaccine matching using monovalent BVS against the in-use vaccine strain IND63/1972 in 2D-VNT. Results showed that all four isolates of G-IX and one isolate of G-VIII exhibited an r value >0.3 with the vaccine strain (Table 4).

**Table 4.** One-way Antigenic relationship values of FMDV serotype Asia1 isolates from 2020 to 2023. All five isolates showed a good antigenic match (r value > 0.3) with the currently used Indian vaccine strain IND63/1972.

S No	Isolate ID	r-value	Genetic Group
1	As/ICFMD1/2020	0.861	Group-IX
2	As/ICFMD 4/2020	0.581	Group-IX
3	As/CFMD8/2020	0.623	Group-IX
4	As/ICFMD120/2021	0.532	Group-IX
5	As/ICFMD 45/2023	0.549	Group-VIII

## 4. Discussion

FMDV serotype Asia1 has been intermittently associated with sporadic outbreaks in India. Between 2011 and 2020, serotype Asia1 was implicated in 5% of the total FMD outbreaks recorded in the country, with yearly variations ranging from 0 to 24.5% (Subramaniam et al. 2022). Notably, serotype Asia1 was linked to numerous outbreaks in 2011 (*n* = 85) and 2012 (*n* = 52). However, the incidence of serotype Asia1 has decreased since then. In 2016 and 2019, serotype Asia1 was not reported from any parts of the country. Geographically, serotype Asia1 exhibits a higher prevalence in the eastern, northeastern, and western parts of the country. Globally, isolates of serotype Asia1 exhibit lower genetic diversity and are typically grouped within a single topotype known as the ‘ASIA’ topotype. Post-2003, isolates of serotype Asia1 have been classified into nine genetic groups within the ASIA topotype, designated as G-I to G-IX. The emergence of G-IX, a novel genetic group initially identified in Bangladesh, was first documented in India in 2020 on an organized farm in the state of Tamil Nadu (Subramaniam et al. 2020). Subsequent outbreaks caused by serotype Asia1 were recorded in Tamil Nadu, Jammu & Kashmir, and Gujarat between 2020 and 2023. In the current study, five isolates collected from these outbreaks were sequenced at the VP1 region to assess the current status of G-IX. The isolates from Tamil Nadu and Jammu & Kashmir were identified as belonging to G-IX, indicating the sustained presence and potential long-distance spread of this group within India. To date, G-IX has only been confirmed in Bangladesh and India. The isolate from Gujarat was grouped with G-VIII, which was exclusively circulating in India between 2005 and 2019 and had not been recorded in the country for the past four years since 2019. The re-emergence of G-VIII after a prolonged period of absence is unforeseen, as the evolution of FMDV typically involves lineage turnover, wherein older lineages or genetic groups are replaced by newer ones. However, sporadic reappearances of previous strains have been reported, such as in the case of serotype C in 2004/2005’ (Paton et al. 2021). In this study, isolates were sequenced at the cell culture passage level 4, where there is a possibility of selecting subpopulations. Given that some samples are closely related both spatially and temporally, although the impact may be minor in this nationwide study, it cannot be entirely discounted

**Figure 3.**
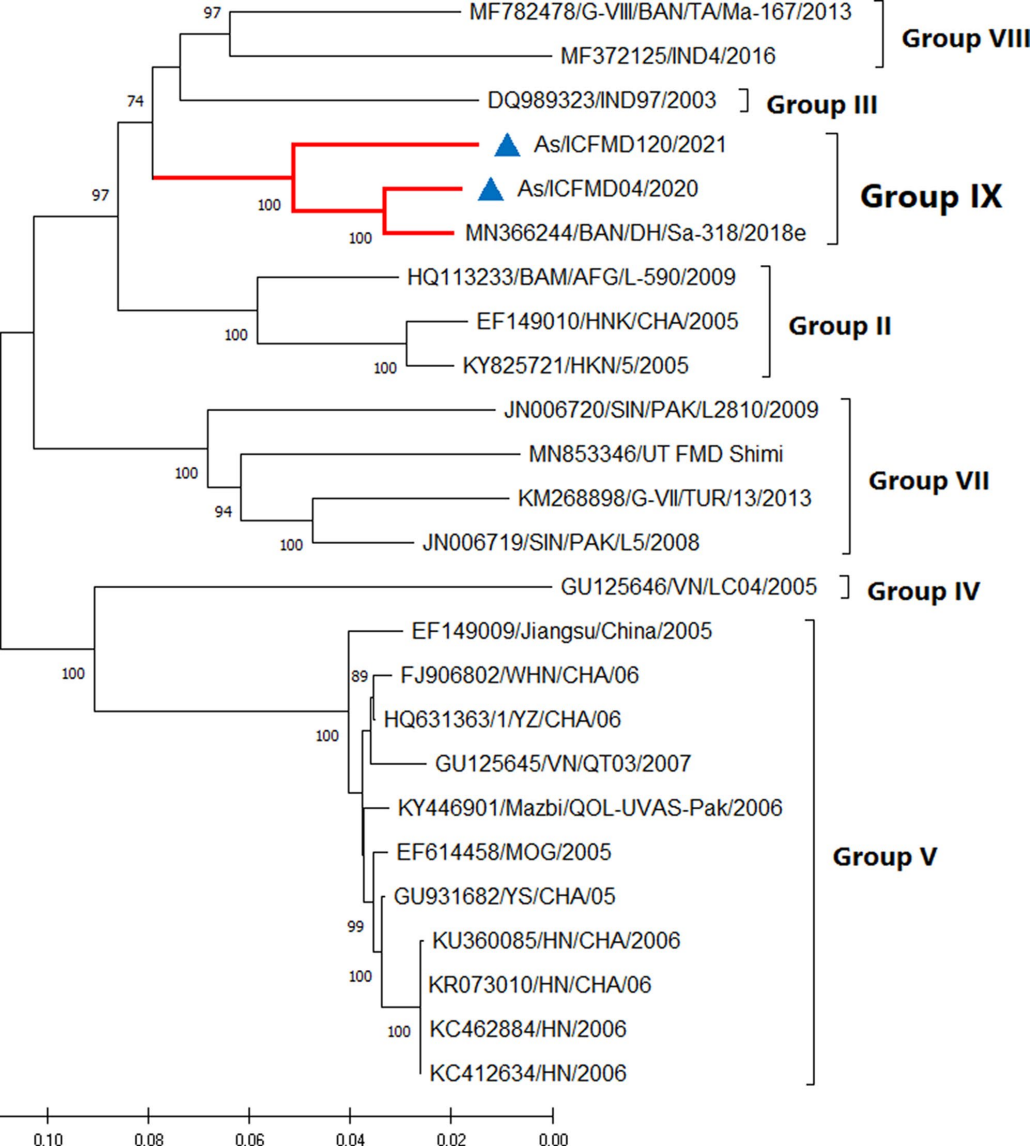
Maximum Likelihood phylogenetic tree based on the complete ORF illustrates various genetic groups of serotype Asia1. The isolates of G-IX originating from India, sequenced in this study, are denoted by filled blue triangles. The percentage of bootstraps (out of 1000) supporting each clade is indicated. Branch lengths are measured in substitutions per site.

The complete coding region sequences of the two isolates of G-IX have been characterized for the first time. The length of the ORF of the two G-IX isolates was 6990 nts, encoding 2329 amino acids, without any deletions or insertions. Critical motifs in the non-structural proteins (NSP) previously described (Mohapatra et al. 2008) were found to be fully conserved. Four independent antigenic sites (I, II, IV, and V) were identified in FMDV serotype Asia1, with critical residues at positions VP1-140, 141, and 142 (Site I), VP2-62, 72, 74, 77, and 79 (Site II), VP3-58 and 59 (Site IV), and VP3-218 (Site V) (Grazioli et al. 2013). Residues critical for antigenic sites on the capsid region were mostly conserved compared to the vaccine strain, except for conservative changes at three positions. The ML phylogeny based on the complete ORF revealed clustering of isolates in their respective groups, as identified in the VP1 region-based phylogeny, with the exception of the placement of groups in the tree topology (Figure 3). For example, G-III and G-VIII, which were distantly placed in the VP1 tree, were more proximally together in the ORF-based tree. Differences were observed in the topologies of the phylogenetic trees based on the L, P1, P2, and P3 regions. Phylogenetic analysis based on VP1 supports the hypothesis that G-VIII and G-IX originated from a common ancestor, as speculated earlier (Subramaniam et al. 2022). This notion was further supported by phylogenetic analysis based on the entire capsid region (P1) and by a high bootstrap value of >70% (data not shown). This incongruence in the clustering pattern is attributed to suspected recombination, a phenomenon commonly observed in FMDV. Recombination plays an important role in the evolution and genetic variation of FMDV and is readily found in many FMDV genomes. Frequent recombination in the FMDV genome, especially in the NSP region, has been documented. Slippage of RNA polymerase in the region with high sequence homology is speculated to be an important reason for recombination. However, recombination was not detected in any of the G-IX isolates analyzed in the current study.

The selection pressure analyses indicated that none of the codon positions in the capsid region were subject to pervasive positive selection pressure. This reduced selection pressure observed in serotype Asia1 may contribute in part to the comparatively lower diversity experienced within this serotype. The diminished positive selection pressure could be attributed to the episodic nature of positive selection events, occurring amidst a backdrop of robust purifying selection that governs many evolutionary trajectories of the virus (Mohapatra et al. 2008). To support this notion, several codon positions, particularly in the capsid region, were identified as being subject to episodic positive selection pressure in the current analyses. It is notable that certain codons undergo purifying selection for the majority of their evolutionary history, punctuated by bursts of intense positive selection. Mutations at these sites may undergo transient positive selection, succeeded by purifying selection to uphold the change, thereby likely playing a pivotal role in adaptive evolution (Murrell et al. 2012).

In the present analyses, an amino acid insertion was observed in the VP1 region of a G-VIII isolate from Gujarat collected in 2023. Previous study has shown a comparable codon insertion in two Asia1 isolates obtained from Madhya Pradesh and Odisha in 2001 (IND321/2001) and 2002 (IND98/2003), respectively (Subramaniam et al. 2013), resulting in a VP1 protein of 212 amino acids long. Isoleucine was added in IND321/2001, while threonine was inserted in IND98/2003 and ICFMD45/2023. The biological implications of these indels are not known, and they seem to be unstable or generate viruses with lower fitness. The insertion found in the two Asia1 isolates collected during 2001 and 2002 has not been detected in any of the isolates except the one sampled in 2023, suggesting their fleeting existence in the virus population during the course of evolution. The observed insertion was speculated to be due to the spatial proximity of certain loops or regions of the genome, which might have favored intra-molecular strand switching by the 3D polymerase, resulting in the origin of a viable progeny virion (Sanyal et al. 2004). The loop regions (such as the βB-βC and βG-βH loops) are structurally disordered and heterogeneous, and can tolerate changes with a minimum loss of function (Grubman & Baxt, 2004). Earlier, a codon deletion at the 44^th^ position of serotype Asia1 has been reported in a particular genetic group, which includes the prototypic strain PAK1/54 and the Indian vaccine strain IND63/1972 (Mohapatra et al. 2004). This genetic group was in circulation during 1964–2000 and was never recorded after 2000. The deletion was located in the βB strand of the βB-βC loop, which is considered to be antigenically critical in other FMDV serotypes.

The effectiveness of the vaccination-based control program is complicated by the emergence of antigenic variants in the field. Determining the antigenic relationship of field isolates with the vaccine strains is an essential requirement to implement appropriate vaccine strains. Vaccine matching, although routine, becomes more important whenever there is the emergence of new genetic groups. Though the isolates of the G-IX group showed considerable genetic distance, all of them were found to have a good antigenic match with the currently used Indian vaccine strain, IND63/1972. In addition, the isolate with a codon insertion in the antigenically critical βG-βH loop also revealed a good antigenic match. In general, serotype Asia1 is considered to be relatively stable antigenically, and the vaccine strain IND63/1972 has been used in the vaccine formulation for more than two decades in India. Vaccine matching data suggest that the currently used Indian vaccine strain showed good antigenic coverage towards both G-VIII and G-IX lineages circulating in India.

## 5. Conclusion

FMDV serotype Asia1 is a potential candidate for elimination from India due to its low prevalence rate, geographically limited circulation, and genetic and antigenic stability. The complete coding region sequences of G-IX isolates recovered from naturally infected cattle have been deciphered for the first time to our knowledge. The investigations showed that the genome has not undergone significant alteration, and no evidence of insertions or deletions in the polyprotein coding region was detected. The results of the vaccine matching studies confirm that the Indian vaccine strain IND63/1972, which is currently in use, is suitable for containing outbreaks caused by G-IX viruses.

## Data Availability

All required data are available as texts and figures in main text of the article. The sequence data sets generated during this research are publicly available at NCBI GenBank.
